# Attitudes of medical doctors, nurses and paramedics towards death

**DOI:** 10.3389/fpubh.2025.1655217

**Published:** 2025-12-04

**Authors:** Krzysztof Zdziarski, Paulina Zabielska, Katarzyna Karakiewicz-Krawczyk, Anna Knyszyńska, Anna Landowska, Marek Landowski, Beata Karakiewicz

**Affiliations:** 1 Subdepartment of Social Medicine and Public Health, Department of Social Medicine, Pomeranian Medical University in Szczecin, Szczecin, Poland; 2 Department of Clinical Nursing, Pomeranian Medical University in Szczecin, Szczecin, Poland; 3 Department of Humanities and Occupational Therapy, Pomeranian Medical University in Szczecin, Szczecin, Poland; 4 Institute of Spatial Management and Socio-Economic Geography, University of Szczecin, Szczecin, Poland; 5 Independent Research and Biostatistics Laboratory, Department of Social Medicine, Pomeranian Medical University in Szczecin, Szczecin, Poland

**Keywords:** death, attitudes, doctor, nurse, paramedic, patient, Poland

## Abstract

**Introduction:**

The phenomenon and experience of death are fundamental aspects of human existence, explored and presented across diverse contexts. The issue of attitudes to death is a very important element in the interactions taking place between medical personnel and patients. The aim of this study was to provide an insight into the attitudes of doctors, nurses and paramedics towards death.

**Method:**

The study was conducted using the Questionnaire of Attitudes Towards Death, which was completed by 320 respondents.

**Results:**

Results from the survey show that the most concrete existential and spiritual attitudes towards death are presented by paramedics compared to the group of medical doctors and nurses.

**Conclusion:**

The problem described is part of a broad discussion on attitudes to death and provides an opportunity for reflection on this phenomenon, especially in the perspective of modern medical technologies, which in many situations limit interactions with patients. Our findings suggest that professional role significantly shapes these attitudes, with paramedics exhibiting the strongest spiritual and escape-oriented acceptance, while physicians demonstrate the most detached perspective.

## 1 Introduction

Death is a process, a phenomenon, a state, an experience accompanied by an emotional attitude: negative, neutral or positive. Synonyms of the term are: dying, end of life, descent, departure. Most of these terms are pejorative, which may support the view that humans have a permanent fear of death. According to psychologists, thanatic anxiety is the most emotionally embedded in the human psyche and has the strongest potential for impact. Researchers are attempting to systematize attitudes towards dying in order to assist those in the medical profession. A review of the literature shows that there are different theories describing the problem of dying. Death is one of the most difficult topics subjected to reflection by thinkers, experts in various scientific fields and ordinary people. Reflection on death is a problem discussed in philosophical, bioethical, theological, medical, legal and social spaces (1).

The literature suggests that starting end-of-life discussions early has a positive impact on the patient’s involvement in dying. Furthermore, it is believed that doctors are ambassadors of clinical knowledge and trusted advocates for patients. It is therefore advisable that they have conversations with patients early in the dying process (2). Doctors, nurses, paramedics face the loss of patients on a daily basis, which affects their emotions, attitudes and the way they do their job. It is important to note that the process of death physically affects not only the dying person, but also has a psychological impact on their caregivers, especially health professionals (3). Understanding the attitudes of health services towards death becomes crucial not only for their own wellbeing, but also for the quality of care they offer to patients and their families. More recently, the COVID-19 pandemic has changed people’s overall view of death and, as a result, provided the impetus for a new view of health and life. Studies indicate that 131 million people died worldwide from various causes in 2020 and 2021, 15.9 million of them due to the COVID-19 pandemic (4). The consequence of the pandemic was a global public health crisis and anxiety and fear of death among the general public and health professionals in particular (5, 6).

The TMT theory also shows the practice of using defence mechanisms depending on whether thoughts about death are conscious or unconscious. According to this dual-process model, when a person’s thoughts about death are conscious, they engage in proximal defence, which involves suppressing these thoughts (e.g., I am not in a high-risk group, so everything will be fine). However, when thoughts of death leave consciousness, distal defence mechanisms are activated, which consist of strengthening defences by supporting cultural worldviews or increasing personal self-esteem (7).

Scientific research based on Terror Management Theory (TMT) shows that the awareness of mortality salience among medical staff in the context of COVID-19 in their relationships with their loved ones increases their defence mechanisms. As a result, this leads to increased interaction and reinforcement of pro-social behaviour. The results of the study show that relational support has a positive effect on reducing the fear of death among medical professionals, even in conditions of high risk to their health. Consequently, this confirms the thesis that strong social support is crucial for mental health and generates pro-social behaviour in overcoming the pandemic (8).

The structure of Terror Management Theory also allows us to see other elements that influence the activation of cultural worldview, thus broadening the areas of research. Research in this area shows that cultural worldview, activated in the distal defence of fear of death, is not only based on modifications in self-esteem. It was tested whether medical personnel reflecting on death would activate a buffer causing higher self-esteem, mood changes, agency and a sense of community. However, no such effect was observed. The only factor that caused changes in this regard was the religious attitude of the respondents participating in the experiment. Other variables, such as age, length of service, income level and membership of a particular professional group, had an impact on self-esteem and mood, but did not interact with exposure to content that increased the frequency of thoughts about death (9). Other studies indicate that cultural systems can act as buffers against death anxiety for their members in diverse existential situations. For example, loose cultures may provide generally available buffers against anxiety, while tight cultures, through extensive social cooperation, alleviate personal anxiety in the face of collective threats. These observations are interesting because previous TMT research has focused on pro-social behaviour related only to personal reminders of death. This opens up a new area of research emphasising the importance of studying collective reminders of death, such as natural disasters and public health crises (10).

The aim of the research undertaken is to present and analyse the attitudes of doctors, nurses and paramedics towards death. In the perspective of the widely understood problem of death, the own research undertaken, concerning the attitudes of the medical professions towards this phenomenon, provides an opportunity to present this problem in the perspective of five spaces: Fear of Death—FD, Death Avoidance—DA, Neutral Acceptance—NA, Approach Acceptance—AA, Escape Acceptance—EA. The survey of attitudes using a standardised tool, the DAP-R-PL, is one way of approximating attitudes towards death and provides a subjective grasp of the problem addressed. The participation of medical professionals in the survey confirms their involvement in death-related issues and may generate positive interactions with dying patients in the future.

## 2 Materials and methods

Study participants were purposively selected, were informed that the study was anonymous, consented to participate and completed the online survey questionnaire. It should be added that the purposeful selection resulted from the research problem, which defined the medical professionals who would participate in the study. The questionnaires were addressed directly to doctors, nurses and paramedics in order to avoid random respondents. The study involved 320 respondents from Poland, including 110 doctors, 93 nurses and 117 paramedics. In the group of doctors, 63% were women, in the group of nurses 90%, while in the group of paramedics the percentage was 27%. Paramedics have the highest average age of 38.85 years, followed by doctors at 32.21 years and nurses at 24.09 years. In each group, the mean age of women is lower than that of men. Detailed results of the age parameters of the respondents are shown in Table 1.

**Table 1:** Age characteristics of respondents participating in the DAP-R survey.

Variable	Medical doctors	Nurses	Paramedics
All (*N*)	110	93	117
Age min–max	25–50	22–37	20–58
Age *M ± SD*	32.21 ± 5.55	24.09 ± 2.62	38.85 ± 8.67
Female (*N*)	69	84	32
Age min–max	25–45	22–37	20–58
Age *M ± SD*	31.43 ± 4.46	23.98 ± 2.39	36.25 ± 7.20
Male (*N*)	41	9	85
Age min–max	27–50	22–35	20–58
Age *M ± SD*	33.51 ± 6.88	25.11 ± 4.26	39.84 ± 9

M, mean; SD, standard deviation.

In addition to basic sociodemographic data (gender, age, place of residence), the survey questionnaire contained 32 statements regarding attitudes towards death from the DAP-R-PL. The answers to the statements were given on a scale from 1 to 7, where 7 means: I strongly agree, 6—I agree, 5—I rather agree, 4—it is difficult to say, 3—I rather disagree, 2—I disagree, 1—I strongly disagree. The answer key with appropriately assigned points includes 5 factors:

1. Fear of Death (FD), which includes 7 statements (contained in questions: 1, 2, 7, 18, 20, 21, 32): death is undoubtedly an unpleasant experience, the prospect of my death makes me anxious, it worries me, that death is inevitable, I am very afraid of death, I am terrified that death will mean the end of everything I know, I am worried about the uncertainty of what will happen after death.
2. Avoiding Death (DA), which consists of 5 statements (from questions: 3, 10, 12, 19, 26): I avoid the thought of death at all costs, whenever the thought of death comes to my mind, I try to push it away, I try not to think about death, I completely avoid thinking about death, I try not to have anything to do with the subject of death.
3. Neutral Acceptance of Death (NA), which includes the statements (assigned to questions: 6, 14, 17, 24, 30): death should be seen as a natural, undeniable and inevitable event, death is a natural aspect of life, I am not afraid of death but I am not waiting for it, death is part of life as a process, death is neither good nor bad.
4. Acceptance of Death (AA), which includes the answers (from questions: 4, 8, 13, 15, 16, 22, 25, 27, 28, 31): I believe that after death I will be in heaven, death is connected with entering in a state of the greatest satisfaction, I believe that heaven will be a much better place than this world, death is union with God and eternal happiness, death brings the promise of a new and wonderful life, after death I look forward to meeting those I love again, I see death as a transition to eternal and blessed place, death frees the soul, the only thing that gives me relief in the face of death is faith in life after death, I look forward to a new life after death.
5. Escape from Death (AE), which includes the statements (from questions: 5, 9, 11, 23, 29): Death will end all my problems, death is an escape from this cruel world, death is salvation from pain and suffering, death is a release from earthly suffering, I see death as a release from the burden of life (11). The following statistical tests were used to analyse the results of the DAP-R questionnaire: Shapiro–Wilk test, Mann–Whitney U test, chi-square test. To test the reliability of the survey, Cronbach’s alpha coefficient was used. Basic statistical measures and graphical presentation of the results were also used.

In this study, age and gender were included as control variables, as research indicates that these socio-demographic factors influence attitudes towards death. Age plays a key role because awareness of the inevitability of death changes with age, reflection on life and its end intensifies, and fear and acceptance of this phenomenon also change. Younger people often have a different perception of death than middle-aged or older people, who think about the end of life more often and may experience greater anxiety about its proximity. Gender is also important, as studies have shown that women think about death more often than men, show greater thanatic anxiety, and also exhibit different mechanisms for coping with this anxiety (12–15).

## 3 Results

Using the Shapiro–Wilk statistical test performed, it was shown that none of the distributions approximated a normal distribution at a significance level of 0.05. The reliability of the survey was examined using the Cronbach’s alpha coefficient. Cronbach’s alpha coefficient for the entire survey is 0.854, while for the individual DAP-R questionnaire, it has the following values: Fear of Death (FD) 0.821, Death Avoidance (DA) 0.836, Neutral Acceptance (NA) 0.652, Approach Acceptance (AA) 0.923, and for Escape Acceptance (EA) it is 0.848. All the Cronbach’s alpha values obtained indicate the reliability of the survey data.

Comparing the age of respondents by profession, statistical tests revealed a statistically significant difference between the ages of each professional group. Dividing the respondents by gender also revealed statistically significant differences in the age of respondents within each professional group. Therefore, the youngest group of respondents were nurses (M = 24.09 years old), followed by medical doctors (M = 32.21 years old), and the oldest were paramedics (M = 38.85 years old). The test results are presented in Table 2.

**Table 2:** Comparison of respondents’ age by profession and gender.

Variable	A vs. B		A vs. C		B vs. C	
	Stat	*p*	Stat	*p*	Stat	*p*
Age of all resp.	10.967	< 0.001	−6.546	< 0.001	−11.572	< 0.001
Women’s age	9.645	< 0.001	−4.106	< 0.001	−7.582	< 0.001
Men’s age	3.535	< 0.001	−3.957	< 0.001	−4.337	< 0.001

A, medical doctors; B, nurses; C, paramedics.

The chi-square test was used to determine the relationship between respondents’ occupation and gender and age. The statistical test revealed a statistically significant relationship between gender and respondents’ occupation, as well as between the two age groups and respondents’ occupation, Table 3.

**Table 3:** Chi-square test results.

Variable	Medical doctors	Nurses	Paramedic	chi-sq	df	*p*
Gender						
Women	69	84	32	85.90	2	< 0.001
Men	41	9	85			
Age						
<29 years old	32	84	10	152.58	2	< 0.001
≥29 years old	78	9	107			

The survey results for the individual groups are as follows. The highest mean value for the FD factor was obtained for the nurse group (*M* ± *SD* = 4.42 ± 1.37) and the lowest for the paramedic group (*M* ± *SD* = 4.02 ± 0.9). For the DA factor, the highest mean value was recorded for the nurses (*M* ± *SD* = 3.73 ± 1.53), the lowest for the paramedics (*M* ± *SD* = 2.81 ± 0.96), and for the doctors’ group the value was *M* ± *SD* = 3.41 ± 1.51. The highest values of all factors of the DAP-R questionnaire were obtained for the NA factor for the paramedic group, in this case *M* ± *SD* = 6.18 ± 0.62. The lowest mean value for this factor was recorded for doctors (*M* ± *SD* = 5.43 ± 1.16).

For the nurses group, the mean value for the NA factor was *M* ± *SD* = 5.6 ± 0.9. The mean scores for the AA questionnaire are highest for the paramedic group (*M* ± *SD* = 4.33 ± 1.01), followed by the doctors’ group (*M* ± *SD* = 4.26 ± 1.53) and the nurses group (*M* ± *SD* = 3.92 ± 1.48). For the EA factor, the mean value for the paramedic group is higher than for the other groups and is *M* ± *SD* = 4.87 ± 1.39. For the nurses, the mean EA value is *M* ± *SD* = 3.95 ± 1.49 and is very close to the mean for the physician group (*M* ± *SD* = 3.94 ± 1.54). Detailed results are shown in Table 4.

**Table 4:** Mean and median response scores to the DAP-R questionnaire.

DAP-R	Medical doctors		Nurses		Paramedics	
	*M ± SD*	*Mdn ± QD*	*M ± SD*	*Mdn ± QD*	*M ± SD*	*Mdn ± QD*
FD	4.17 ± 1.45	4.07 ± 1	4.42 ± 1.37	4.29 ± 1.21	4.02 ± 0.9	4 ± 0.64
DA	3.41 ± 1.51	3.2 ± 1	3.73 ± 1.53	3.6 ± 0.9	2.81 ± 0.96	2.8 ± 0.5
NA	5.43 ± 1.16	5.6 ± 0.8	5.6 ± 0.9	5.6 ± 0.7	6.18 ± 0.62	6.4 ± 0.3
AA	4.26 ± 1.53	4.35 ± 0.9	3.92 ± 1.48	4 ± 0.75	4.33 ± 1.01	4.2 ± 0.6
EA	3.94 ± 1.54	4 ± 1.1	3.95 ± 1.49	3.8 ± 1	4.87 ± 1.39	5.2 ± 1.2

FD, fear of death; DA, death avoidance; NA, neutral acceptance; AA, approach acceptance; EA, escape acceptance; M, mean; SD, standard deviation; Mdn, median; QD, quartile deviation.

**Figure 1:**
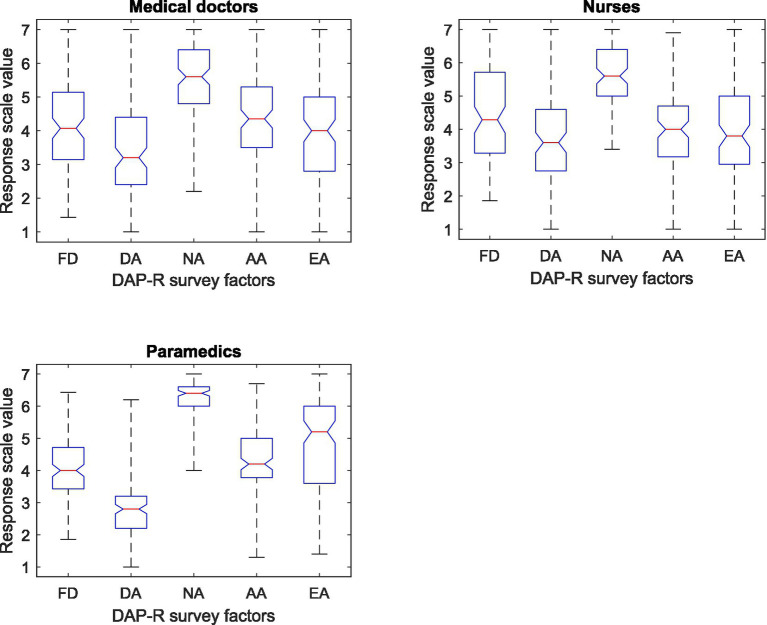
Box plots showing the median, upper and lower quartiles, maximum and minimum values of the DAP-R survey results by DAP-R factor and survey group.

Figure 1 presents box plots with marked median values, lower and upper quartiles, minimum and maximum values for individual DAP-R factors according to the respondent group.

**Figure 2:**
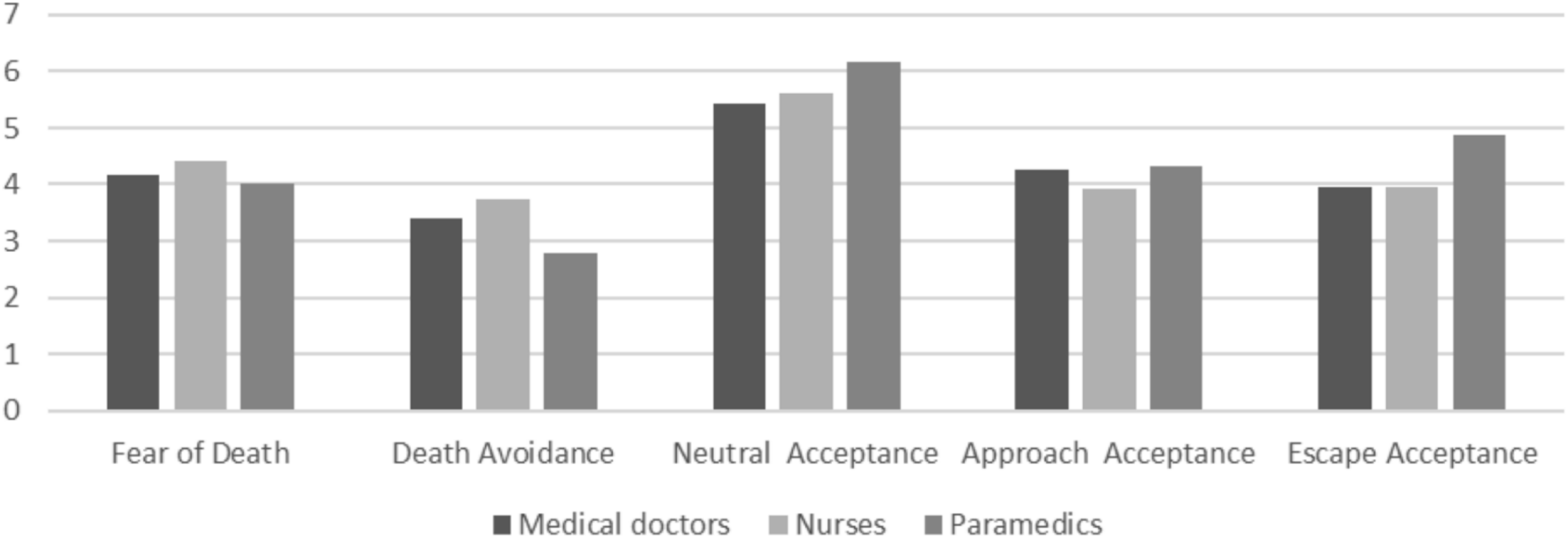
Mean scores for responses to the DAP-R questionnaire.

Figure 2 presents the mean results of the DAP-R questionnaire responses for individual respondent groups.

Statistical tests were conducted to examine differences between DAP-R questionnaire results for different respondent groups. Statistically significant differences were obtained in at least one of the study groups for the DA, NA, AA, and EA factors (*p* < 0.001). Statistical tests examining the relationship between DAP-R questionnaire responses and profession were performed for all respondents and by gender and age. Considering all respondents, the tests showed that for the DA factor, statistically significant differences were obtained in the test result parameters for the paramedic group compared to the other two professional groups. A statistically significant difference can be observed in the median values. For the DA factor, the median value is significantly lower for respondents from the paramedic group. For the NA and EA factors, statistically significant differences were also obtained in the parameters related to the questionnaire results for the paramedic group compared to the other two professional groups. However, in this case, the paramedic group’s median values for the NA and EA factors are statistically significantly higher than for respondents from the medical doctors and nurses groups. For the AA questionnaire, considering the entire group of respondents, a statistically significant difference was obtained between the results of nurses and paramedics. Paramedics demonstrate a higher AA index than nurses. In the case of the questionnaire results for the FD factor, the statistical test did not reveal statistically significant differences in the obtained parameters. Subsequent tests were performed by gender (women, men) and age (under 29 years, 29 years or older). Differences between women and men, as well as by age, were indicated in the results of the DA, NA, AA, and EA questionnaires. For the DA questionnaire, a statistically significant higher median value was obtained for male medical doctors (Mdn = 3.2) than for male paramedics (Mdn = 2.6). For the NA and EA questionnaires, the situation is reversed; male paramedics have a higher median value than medical doctors. For women, statistically significant differences were found in the median values between paramedics and the medical doctors and nurses groups for the NA and EA questionnaires. In both cases, the median values were higher for female paramedics than for women in the other two professional groups. The results for the AA questionnaire indicated a statistically significantly higher median value for female paramedics (Mdn = 4.8) compared to female nurses (Mdn = 4). Analyzing the results by age, among those under 29 years of age, a statistically significant difference was obtained for the DA, NA, and EA questionnaires. A statistically significantly higher median value for DA was obtained for nurses under 29 years of age (Mdn = 3.6) compared to medical doctors (Mdn = 2.5) and paramedics (Mdn = 2.6). In the DA results between the group of doctors and paramedics aged 29 years or older, the statistical test showed that the median DA for medical doctors (Mdn = 3.4) was significantly higher than for paramedics (Mdn = 2.8). The opposite situation occurred in these groups for the results of the NA and EA surveys; here, the rates were significantly higher for paramedics than for medical doctors. Additionally, for the AA questionnaire, a statistically significantly lower median value was observed for nurses (Mdn = 2.6) than for medical doctors (Mdn = 4.65) and paramedics (Mdn = 4.3), the group of people aged 29 years or older. In this age range, a significantly lower median value for nurses (Mdn = 3.4) was also obtained for the EA questionnaire compared to paramedics (Mdn = 5.4). Similarly, in the age group under 29 years, the median score for the NA questionnaire among nurses (Mdn = 5.6) is significantly lower than the median score for paramedics (Mdn = 6.3). However, the results for the EA questionnaire among the group of people under 29 years of age indicate a significantly higher median value for nurses (Mdn = 4) than for medical doctors (Mdn = 3). The results of statistical tests, means, and medians for individual cases are presented in Table 5.

**Table 5:** DAP-R survey results for the entire group of respondents and by gender and age.

DAP-R		A		B		C		A vs. B		A vs. C		B vs. C	
Variable	Group	*N*	M/Mdn	*N*	M/Mdn	*N*	M/Mdn	Z stat	*p*	Z stat	*p*	Z stat	*p*
FD													
Gender	All resp.	110	4.17/4.07	93	4.42/4.29	117	4.02/4	−1.107	0.268	0.465	0.642	1.829	0.067
	Women	69	4.29/4.14	84	4.41/4.29	32	4.33/4.36	−0.427	0.669	−0.314	0.754	0.80	0.956
	Men	41	3.97/4	9	4.49/4.71	85	3.90/3.86	−0.871	0.384	0.018	0.985	1.118	0.264
Age	<29 yo	32	3.88/3.71	84	4.44/4.29	10	3.9/3.89	−1.801	0.072	−0.207	0.836	1.177	0.239
	≥ 9 yo	78	4.29/4.14	9	4.17/3.29	107	4.03/4	0.383	0.702	1.063	0.288	−0.294	0.769
DA													
Gender	All resp.	110	3.41/3.2	93	3.73/3.6	117	2.81/2.8	−1.556	2.120	3.282	<0.01	4.938	<0.001
	Women	69	3.48/3.2	84	3.76/3.6	32	3.25/3	−1.298	0.194	0.566	0.572	1.794	0.073
	Men	41	3.3/3.2	9	3.4/3.8	85	2.64/2.6	−0.354	0.724	2.639	< 0.01	1.632	0.103
Age	<29 yo	32	2.74/2.5	84	3.71/3.6	10	2.76/2.6	−3.187	< 0.01	0.286	0.790	2.324	< 0.05
	≥29 yo	78	3.69/3.4	9	3.89/3.6	107	2.81/2.8	−0.105	0.917	4.250	<0.001	1.440	0.150
NA													
Gender	All resp.	110	5.43/5.6	93	5.6/5.6	117	6.18/6.4	−0.665	0.506	−5.157	<0.001	−4.895	<0.001
	Women	69	5.38/5.6	84	5.58/5.6	32	5.98/6.2	−0.807	0.420	−2.478	< 0.05	−2.156	<0.05
	Men	41	5.51/5.6	9	5.84/5.6	85	6.25/6.4	−0.543	0.587	−3.359	<0.001	−1.510	0.131
Age	<29 yo	32	5.58/5.8	84	5.55/5.6	10	6.14/6.3	0.544	0.587	−1.462	0.144	−2.097	<0.05
	≥29 yo	78	5.37/5.5	9	6.07/6.4	107	6.18/6.4	−1.596	0.111	−4.811	<0.001	0.046	0.963
AA													
Gender	All resp.	110	4.26/4.35	93	3.92/4	117	4.33/4.2	1.884	0.060	0.317	0.751	−2.138	<0.05
	Women	69	4.33/4.6	84	3.99/4	32	4.57/4.8	1.903	0.057	−0.467	0.640	−2.604	<0.01
	Men	41	4.16/4.2	9	3.34/2.6	85	4.24/4.1	1.061	0.289	0.013	0.990	−1.156	0.247
Age	<29 yo	32	3.78/4	84	4.01/4.05	10	3.95/4.1	−0.510	0.610	−0.207	0.836	0.018	0.985
	≥29 yo	78	4.46/4.65	9	3.1/2.6	107	4.37/4.3	2.272	<0.05	0.997	0.319	−2.456	<0.05
EA													
Gender	All resp.	110	3.94/4	93	3.95/3.8	117	4.87/5.2	0.050	0.960	−4.634	<0.001	−4.264	<0.001
	Women	69	3.98/4	84	3.87/3.8	32	4.99/5.6	0.554	0.580	−3.518	<0.001	−3.734	<0.001
	Men	41	3.87/3.6	9	4.69/5.2	85	4.83/5	−0.922	0.357	−3.075	<0.01	0.000	1.000
Age	<29 yo	32	3.29/3	84	4.002/4	10	4.04/3.8	2.366	<0.05	−1.299	0.194	0.006	0.995
	≥29 yo	78	4.2/4.2	9	3.49/3.4	107	4.95/5.4	1.519	0.129	−3.525	<0.001	−2.528	<0.05

A, medical doctors; B, nurses; C, paramedics; FD, Fear of Death; DA, Death Avoidance; NA, Neutral Acceptance; AA, Approach Acceptance; EA, Escape Acceptance; M, mean; Mdn, median.

Further analysis involved examining the statistically significant relationship between DAP-R survey results by occupation, taking into account age and gender. The statistical test revealed a significant difference in median values between women under 29 years of age in the medical doctor group (Mdn = 3.57) and nurses (Mdn = 4.29) for the FD questionnaire. A similar situation occurred for the DA questionnaire results; women under 29 years of age in the medical doctor group (Mdn = 2.7) had a significantly lower median than nurses (Mdn = 3.6). For the DA questionnaire results, a significant difference was also obtained between the responses of men aged 29 or older who were paramedics and those of medical doctors and nurses. In this case, the median result for paramedics (Mdn = 2.8) was significantly lower than that of medical doctors (Mdn = 3.6) and nurses (Mdn = 4.7). A statistically significant difference was also observed among men aged 29 years or older in the responses to the NA questionnaire between paramedics and nurses and medical doctors. A significantly higher median value was obtained for paramedics (Mdn = 6.4) compared to medical doctors (Mdn = 5.6) and male nurses (Mdn = 5.4). Significant differences were also observed in the responses of women aged 29 years or older, with the median for medical doctors (Mdn = 5.4) being significantly lower than the medians for nurses (Mdn = 6.6) and paramedics (Mdn = 6.2). In the responses to the AA questionnaire, a statistically significant difference was observed among women under 29 years of age. In this case, the median for nurses (Mdn = 2.6) was significantly lower than the medians for medical doctors (Mdn = 4.8) and paramedics (Mdn = 4.85). In the EA survey, statistically significant differences in responses were observed among women aged 29 or older between each of the three analyzed professional groups. In this case, the median for medical doctors was Mdn = 4.2, for nurses Mdn = 3.2, and for paramedics Mdn = 5.6. The means, medians, and test results of the DAP-R survey responses by profession, age, and gender are presented in Table 6.

**Table 6:** Mean, median and statistical test results for DAP-R surveys by occupation, taking into account gender and age.

DAP-R		A		B		C		A vs. B		A vs. C		B vs. C	
Age	Gender	*N*	M/Mdn	*N*	M/Mdn	*N*	M/Mdn	Z stat	*p*	Z stat	*p*	Z stat	*p*
FD													
< 29 yo	Women	18	3.71/3.57	77	4.44/4.29	2	4.86/4.86	−1.961	<0.05	−1.323	0.186	−0.593	0.553
	Men	14	4.09/4	7	4.45/4.71	8	3.66/3.71	−0.187	0.852	0.614	0.539	1.273	0.203
≥ 29 yo	Women	51	4.5/4.29	7	4.04/3.29	30	4.29/4.21	0.776	0.438	0.631	0.528	−0.795	0.427
	Men	27	3.9/4	2	4.64/4.64	77	3.93/4	−0.344	0.731	−0.304	0.761	0.172	0.864
DA													
<29 yo	Women	18	2.7/2.7	77	3.77/3.6	2	4.5/4.5	−2.792	<0.01	−1.071	0.284	−0.406	0.685
	Men	14	2.79/2.4	7	3.03/3.4	8	2.33/2.3	−0.448	0.654	0.444	0.657	0.868	0.385
≥29 yo	Women	51	3.75/3.4	7	3.66/2.8	30	3.17/3	0.489	0.625	1.487	0.137	−0.039	0.969
	Men	27	3.57/3.6	2	4.7/4.7	77	2.68/2.8	−1.248	0.212	3.366	<0.001	2.200	<0.05
NA													
< 29 yo	Women	18	5.56/5.8	77	5.52/5.6	2	6.1/6.1	0.280	0.779	−0.693	0.488	−0.967	0.333
	Men	14	5.6/6	7	5.97/6.2	8	6.15/6.3	−0.485	0.628	−0.853	0.394	−0.231	0.817
≥29 yo	Women	51	5.32/5.4	7	6.26/6.6	30	5.97/6.2	−1.993	<0.05	−2.347	<0.05	1.435	0.151
	Men	27	5.46/5.6	2	5.4/5.4	77	6.26/6.4	0.172	0.863	−3.166	<0.01	−1.982	<0.05
AA													
<29 yo	Women	18	3.86/4	77	4.09/4.1	2	4.7/4.7	−0.275	0.783	−1.071	0.284	−1.061	0.289
	Men	14	3.67/3.65	7	3.13/2.6	8	3.76/4	0.709	0.478	0.000	1.000	−0.347	0.728
≥29 yo	Women	51	4.49/4.8	7	2.81/2.6	30	4.56/4.85	2.542	< 0.05	0.152	0.879	−2.947	<0.01
	Men	27	4.41/4.3	2	4.1/4.1	77	4.29/4.1	0.1291	0.897	0.523	0.601	0.000	1.000
EA													
<29 yo	Women	18	3.48/3.4	77	3.95/4	2	4.4/4.4	−1.458	0.145	−0.567	0.571	−0.218	0.827
	Men	14	3.06/2.6	7	4.54/5.2	8	3.95/3.8	−1.417	0.156	−1.229	0.219	0.579	0.563
≥29 yo	Women	51	4.15/4.2	7	3/3.2	30	5.03/5.6	2.005	< 0.05	−2.968	< 0.01	−3.122	<0.01
	Men	27	4.3/4	2	5.2/5.2	77	4.92/5.2	−0.559	0.576	−1.883	0.059	0.437	0.662

A, medical doctors; B, nurses; C, paramedics; FD, Fear of Death; DA, Death Avoidance; NA, Neutral Acceptance; AA, Approach Acceptance; EA, Escape Acceptance; M, mean; Mdn, median.

The correlation between responses for the analysed DAP-R factors for the different groups of respondents was also examined. Among the group of doctors, a statistically significant moderate positive correlation was obtained between responses for the factors FD and DA (r = 0.51, *p* < 0.001) and AA and EA (r = 0.44, *p* < 0.001). The remaining statistically significant correlations are of weak strength and occur for factors such as: FD and NA (r = −0.32, r < 0.001), FD and AA (r = 0.37, *p* < 0.001), FD and EA (r = 0.30, r < 0.01), DA and NA (r = −0.38, *p* < 0.001), DA and AA (r = 0.32, p < 0.001) and DA and EA (r = 0.24, p < 0.001). For nurse respondents, a strong correlation between responses was obtained for factors FD and DA (r = 0.61, 0.001), moderate for factors AA and EA (r = 0.44, *p* < 0.001). A weak statistically significant correlation was detected for factors such as: FD and NA (r = −0.37, p < 0.001), and DA and NA (r = −0.35, p < 0.001). A moderate positive correlation was also found among responses for the paramedic group for the factors FD and DA (r = 0.49, p < 0.001), FD and AA (r = 0.44, p < 0.001) and DA and NA (r = −0.55, p < 0.001). A weak but statistically significant correlation was found between the factors FD and NA (r = −0.37, p < 0.001), FD and EA (r = −0.38, p < 0.001), NA and EA (r = 0.27, *p* < 0.01) and AA and EA (r = −0.2, *p* < 0.05). The detailed correlation results for the DAP-R questionnaire are presented in Table 7.

**Table 7:** Spearman rank correlation for individual DAP-R questionnaires according to the respondent group.

DAP-R	FD	DA	NA	AA	EA
Medical doctors					
FD	1				
DA	0.51***	1			
NA	−0.32***	−0.38***	1		
AA	0.37***	0.32***	−0.05	1	
EA	0.30**	0.24***	−0.01	0.44***	1
Nurses					
FD	1				
DA	0.61***	1			
NA	−0.37***	−0.35***	1		
AA	0.06	0.08	−0.07	1	
EA	−0.17	−0.03	0.14	0.44***	1
Paramedics					
FD	1				
DA	0.49***	1			
NA	−0.37***	−0.55***	1		
AA	0.44***	0.13	−0.16	1	
EA	−0.38***	0.17	0.27**	−0.20*	1

*p < 0.05. **p < 0.01. ***p < 0.001, FD, fear of death; DA, death avoidance; NA, neutral acceptance; AA, approach acceptance; EA, escape acceptance.

## 4 Discussion

Based on the main research tool used, the data obtained on respondents’ attitudes towards death can be presented in five dimensions: Fear of Death—FD, Death Avoidance—DA, Neutral Acceptance—NA, Approach Acceptance—AA, Escape Acceptance—EA.

### 4.1 The fear of death dimension—FD

In this space encompassing attitudes of fear of death, age was a determinant of attitudes towards this phenomenon. Nurses as the youngest group of respondents revealed the greatest fear of death. Although paramedics reported numerically lower mean scores for Fear of Death (FD) than nurses, this difference was not statistically significant (*p* = 0.067). In contrast, doctors, being a slightly younger group in terms of age compared to paramedics, revealed a medium fear of death. The differences between Fear of Death (FD) scores for the groups of doctors, nurses and paramedics are not statistically significant. In the case of young women, it was found that young nurses had a higher fear of death than young female medical doctors. In the remaining cases, gender had no significant impact on fear attitudes towards death. Research by other authors on attitudes towards death, indicated that young female nurses in training, who were characterised by emotional, cognitive and expressive empathy, showed less fear and rational attitudes towards death (16). In contrast, fear of death was highest in nurses working in intensive care and emergency departments (17).

Research highlights that fear of death among ICU and emergency nurses correlates with variables such as workload, patient acuity, and limited resources, all of which can intensify emotional exhaustion and anxiety. Studies also suggest that resilience and coping strategies play a critical role in mitigating death anxiety in these nurses, emphasizing the need for targeted interventions, psychological support, and training to foster emotional resilience and reduce fear (18–20). Other studies conducted in a group of nurses using TMT, aimed at verifying whether contact with older adults affects the level of fear of death and self-esteem, indicated a high level of fear of death and a low level of self-esteem, which were a consequence of the respondents’ high ageism (21).

Based on the results from our own research, it can be seen that in paramedics death does not cause much anxiety and they are less worried about what happens after death. The revealed distancing from death may be a consequence of greater professional experience, including circumstances related to death. It is also possible to see in this group of respondents a process of habituation, during which, as a result of repeated exposure to a constant stimulus, reactions weaken and may fade over time (22). Similar behaviours were observed in paramedics during the COVID-19 pandemic, who were constantly at risk of losing their health and lives (23). Other studies conducted in Iran involving paramedics aged 33–40 report a high level of fear of death. This contrasts with Polish findings, where paramedics tend to exhibit lower death anxiety. These cross-cultural differences highlight a diverse perspective on death-related behaviors, influenced by both the age of respondents and their geographical location. Such variations underscore the importance of considering cultural and demographic contexts when examining attitudes towards death among healthcare professionals. For example, Ghahramanipirsalami found significant death anxiety among prehospital emergency personnel in southern Iran, linked to occupational stress factors, whereas studies in Poland indicate relatively lower fear levels in similar age groups, suggesting that cultural, educational, and systemic factors shape these attitudes distinctly (24, 25). Based on the literature, it can be seen that exposure to content that increases thoughts of death causes changes in the behaviour of doctors, nurses and paramedics. in areas such as self-esteem, mood, sense of agency and community (9). In this situation, the scientific view that death anxiety is a natural multidimensional cognitive, emotional and experiential process is validated (26). Other studies on fear of death using TMT have shown that medical workers’ exposure to information about COVID-19 was positively associated with their fear of death and reflection on death, which in turn led to frequent withdrawals from medical work (27). Other TMT-based studies have shown that satisfaction with interpersonal relationships effectively reduces fear of death. In addition, healthcare workers with strong social support showed greater satisfaction with personal relationships (28). Studies based on manipulating self-esteem to examine fear of death are also interesting. The results indicated that a threat to self-esteem generated greater fear of death. In contrast, higher self-esteem alleviated this attitude (29). Fear of death among doctors, nurses, and paramedics is a complex issue influenced by age and cultural background. Cross-cultural studies demonstrate that cultural norms and beliefs shape how healthcare professionals perceive and manage death anxiety. Study among healthcare professionals in Spain found that lack of psychological support during the COVID-19 pandemic raised death anxiety levels, especially for younger staff (30). In China, research on medical interns showed that women and those from families with less open discussion about death experienced higher death anxiety, while attending more funerals correlated with more neutral acceptance of death (31). Among emergency medical technicians in Iran, nearly half reported severe death anxiety, illustrating the emotional burden faced by younger, frontline workers (32). These findings underscore the need for age- and culture-sensitive psychological interventions and training to build resilience and reduce fear among healthcare workers across diverse settings.

### 4.2 The death avoidance dimension—DA

A statistically significant difference in the presented Death Avoidance (DA) scores was observed between the groups of doctors and paramedics and nurses and paramedics. Paramedics had significantly lower avoidance than both doctors and nurses. The results of our own research indicate that doctors and nurses are the groups of respondents who most often reject thoughts of death and try not to have anything to do with the subject. It should also be noted that in our study we found that in the group of women under 29 years of age, young nurses showed a higher DA index than young women medical doctors. For example, a study by Üstükuş and Eskimez showed that as nurses’ death anxiety increased, their avoidance of dying patients actually decreased, indicating a complex relationship between anxiety and coping through distancing behaviors (33). In contrast, paramedics are the most familiar with the topic and do not push away topics related to thinking about death. Paramedics often show greater resistance to stress and the ability to cope with difficult situations, which is essential in their life-saving work. This attitude may stem from their strong motivation, commitment and willingness to seek professional challenges, which translates into a more mature and constructive approach to death. Unlike nurses and doctors, paramedics are better able to cope with the emotional aspects of dealing with death, which is invaluable in the context of caring for critically ill and terminally ill patients (34). Doctors are close to the attitudes of nurses in their opinions. When considering the data collected in terms of the gender of the respondents, no statistically significant differences were noted. Other studies indicate that healthcare professionals working in oncology and palliative care are not afraid of conversations related to the topic of death avoidance (35). Observations can be found in the literature that indicate that nurses who received good education about death acquired the skills to talk about death with dying patients (36). Research reports indicate the need to educate medical staff in the perspective of the death phenomenon, because, as studies show, some nurses did not receive any support from doctors, but only from fellow nurses (37).

The above view corresponds with the thesis of researchers who affirm that positive attitudes should be generated among medical professionals to nullify death-avoidance attitudes (38, 39). Other implications highlight the need to develop interventions focusing on palliative skills-based training and emotional support for carers of people in a terminal state, in order to counteract the fatigue in them that generates negative attitudes towards death (40). In the presented perspective of death avoidance, it is important to mention the avoidance of death through the use of anaesthesia as a causal tool for dying peacefully, while at the same time escaping from thinking about this phenomenon. The research confirms that positive connotations referred to the perception of anaesthesia as an easier pathway, and concerns related to depriving patients of the awareness of dying (41, 42). In the space of the death avoidance phenomenon, the attitudes of Chinese nurses are interesting, revealing that their society has a culture of death avoidance and denial. Influenced by these characteristic socio-cultural views, nurses often find it difficult to with caring for dying patients and coping with death (43). Considering one’s worldview as a determinant of attitudes towards death, it is worth recalling the Terror Management Theory (TMT), which assumes that thoughts of death, by triggering fear of self-destruction, motivate the defence of one’s personal cultural worldview. Researchers of uncertainty suggest that thoughts of death trigger feelings of uncertainty, which motivate people to defend their worldview (44). Scientific reports emphasise that building self-esteem by adhering to worldview standards helps to alleviate fears of death (45).

### 4.3 Neutral acceptance dimension—NA

Another dimension concerning attitudes towards death is related to the neutral approach to death (NA) and the recognition of death as part of the human life process. While paramedics demonstrated a statistically significant higher Neutral Acceptance (NA) score than both other groups, no significant difference was found between medical doctors and nurses. As for gender, in the group of paramedics, a statistically significant difference was observed among men age 29 or older who showed a higher degree of neutral acceptance of death (NA) compared to medical doctors and nurses. However, female medical doctors aged 29 or older had lower NA scores than nurses and paramedics. The work of a paramedic involves frequent contact with death and critical situations, which necessitates the development of skills for emotional distancing and accepting death as a natural part of life. Men in this group often demonstrate greater stress resilience and a pragmatic approach to the issue of death, which is essential in the fast-paced environment of emergency medical services. Additionally, cultural and social norms may influence men to accept death more neutrally, avoiding excessive emotionality and acknowledging death as an inherent and factual aspect of their profession. This attitude enables them to better manage the pressures associated with saving lives and making difficult decisions, including those related to ending resuscitation or providing palliative care (43). Doctors, on the other hand, pay the least attention to this research factor. Results from other studies show that the natural acceptance of death by health professionals is helped by: personal acceptance of the death phenomenon or avoidance of thinking about death and the use of personal communication with patients (46).

Furthermore, scientific reports indicate that people with a low natural acceptance of death are more likely to avoid contact and have difficulty communicating with dying patients (44). The prospect of a neutral approach to death is associated with the problem of ineffectiveness, which is understood as the use of treatments that do not bring significant benefits to patients. Research findings on this issue show that nurses focused mainly on agony, suffering and risk, while doctors paid more attention to the hope of recovery. In general, both medical groups were concerned about different treatment methods: nurses about the invasiveness of treatment, and doctors about the excessive medicalisation of death. They emphasised the right of patients to decide on the end of their life and to freely express their will when they are terminally ill, not to be resuscitated (47).

### 4.4 Approach acceptance—AA

The space of acceptance of death encompasses the spiritual and religious sphere of a person, which is part of the general definition of health and reveals a personal axiology expressing inner feelings related to dying. The strength of faith or religious denomination varies from person to person and influences opinions on life and death, including medical topics related to palliative and emergency medicine (48). Some religious people, for example evangelical Christians in the USA, are more likely to request life-prolonging procedures than non-religious people. Furthermore, Protestants are more likely to agree to euthanasia than Catholics (49). The data obtained from our own research indicates that paramedics and doctors are the most spiritually oriented towards experiencing death, and nurses are less so. Taking into account the gender of the respondents, it should be noted that women working as paramedics are the most likely to reveal their spiritual acceptance of death, and men in the nursing profession are the least likely.

This thesis corresponds to a large extent with studies which show that over 60% of paramedics who consider themselves to be moderately religious and 20% who regularly use religious values in their daily life, claim that life support is unethical (50). In the age of globalisation, different cultures and religions, it is interesting to look at the approach to death among followers of Islam. The Islamic code indicates that any act of taking a life is forbidden. Therefore, Muslims do not have the right to end their own lives. Withholding or withdrawing medical treatment is only permissible if the patient suffers from an incurable fatal illness (51). In the discussed religious approach to accepting death, it is interesting to note that studies have shown that nurses practising a particular religion are able to accept the death of a patient positively, while those who do not practise religious principles have found it difficult to accept death (52). Other studies show a wide variety of attitudes among Jews towards the end of life and the use of voluntary death (53). In the literature, one can also find data that imply that religion provides hope, which reduces the fear of death, more in older people than in younger ones (54). Scientific research points to the positive psychological significance of faith as a way of coping with fears related to death (55). Furthermore, studies conducted using TMT have shown that religious attitudes influence effective defence against negative acceptance of thoughts about death (9). Other scientific reports (using TMT) explain how fear of illness and death is controlled through religion. The results of these studies indicate a positive relationship between religious attitudes and fear of death and fear of COVID-19 (56). From the perspective of Terror Management Theory (TMT), religion can support the management of fear caused by human awareness of death by providing a sense of psychological security and alleviating fear of death (57).

### 4.5 Escape acceptance—EA

The respondents also expressed their opinion on escaping death, understood as freedom from suffering and the burden of life. Paramedics had significantly higher Escape Acceptance (EA) than both doctors and nurses. Thus, paramedics are most in agreement with the thesis that death solves all human problems and is a deliverance from pain and suffering. This perspective may result from the unique nature of their work, which exposes them regularly to life-and-death situations. For paramedics, death can be seen not only as an end but also as a form of relief for patients enduring significant physical or emotional suffering. Such a viewpoint may help paramedics develop emotional resilience necessary for their demanding roles, allowing them to approach death with a more pragmatic and accepting attitude. This acceptance can assist them in managing the challenges of their profession, including making difficult decisions related to end-of-life care. Their experience often leads to the development of cognitive frameworks that help them cope effectively with the emotional impact of patient deaths, supporting balanced professional conduct despite the emotional toll (58). Doctors and nurses are less likely to share this view. When it comes to the gender of the respondents, female paramedics are the most likely to agree that death is a release from pain and suffering and will end all human problems. In view of this opinion, the principle of palliative sedation and analgesia, known as the principle of double effect, practised by some medics, can be recalled. This doctrine is used to justify an action that has a good effect: the relief of suffering, while at the same time having a bad effect: hastening death (59). In scientific literature, there is an ongoing discussion about whether and how this doctrine can be applied in practice (60). Palliative sedation and analgesia are often criticised by those who claim that it is a process of hastening or causing death. There are also experts in the field of palliative care who disagree with this principle (61).

The literature also includes observations of doctors and nurses who have a direct influence on end-of-life decisions. The results show that doctors tended to strive to meet the family’s needs, while nurses tended to act on behalf of the patient and what they interpreted as being best for the patient (62). Another study showed that the wishes of a family member should be clearly distinguished from the quality of the patient’s end of life, as the two aims are completely opposite (63). In terms of using death as a release from suffering and a release from the burden of life, studies describing an experiment conducted among doctors who were subjected to a desensitisation process before death are interesting. The results showed only partial desensitisation with regard to egoism and altruism (64). From an existential perspective, this is a positive finding, as negative attitudes towards dying people can lead to inadequate care (35). The researchers also emphasise the need for end-of-life training for trainee doctors. Studies show that trainees who showed moderate fear of death had positive interactions with dying patients and did not avoid topics related to death. In contrast, people with a higher level of fear of death tended to avoid contact and had greater difficulty communicating with the dying (47). Other reports confirm that knowledge of palliative care can improve the quality of care for patients at the end of life and the perception of one’s own death (65). Also interesting are the results of studies using TMT, which emphasise the value of communicating information about death and how other people think about the end of life in order to control and reduce the fear of death (66).

Taking into account the data from our own research, it should be emphasised that the behaviours presented show only a fragment of the condition of medical personnel after the COVID-19 pandemic. The study of attitudes towards death is the subject of numerous studies that identify new problems and challenges in protecting the mental health of healthcare professionals. Researchers reveal that interactions with family or colleagues have a significant impact on improving the psychosocial condition of medical services (67). A review of the literature consolidates the experiences of healthcare workers in their interactions with the dying, emphasising, among other things, the importance of team support and self-care for all medical staff, regardless of their level of involvement (68). It should be noted that healthcare workers experience many positive and negative emotions that shape their personal view of death. In addition, emotions also generate the inner strength to carry out tasks undertaken for the dying or cause withdrawal as a result of difficult existential experiences in contact with death (69). It should be added that after the COVID-19 pandemic, the psychological state of healthcare workers is significantly burdened. They experience higher levels of depression, anxiety and stress. The pandemic has brought new mental health challenges that may adversely affect the motivation, willingness and openness of healthcare workers in the coming years (70, 71). It has also had a lasting impact on the mental well-being of medical professionals. It is advisable that social and health policies be directed towards supporting healthcare workers and improving their mental health (72). The study results indicate the need for training programs focused on death communication and anxiety management for young nurses, as well as spiritual debriefing sessions for paramedics. Implementing training that develops psychosocial and spiritual competencies can strengthen the psychological resilience of healthcare personnel and improve the quality of patient care.

## 5 Study limitations

This study has certain limitations:

1. Lack of Professional Experience Data: Age is used as a proxy, but years of clinical experience is a far stronger predictor of death attitudes. Future studies should measure this directly.
2. Lack of Religious Affiliation Data: Given the centrality of the Approach Acceptance (AA) factor and its link to spirituality/religion, asking respondents about their religious beliefs or practices would have provided crucial context for interpreting AA scores (especially for paramedics).
3. Specialization Unknown: Not disclosed were doctors oncologists, were nurses in ICU, were paramedics in urban EMS? These settings drastically influence exposure to death.
4. Sampling Method: It was convenience, which constitutes a limitation regarding generalizability beyond the sampled institutions.
5. Cultural Specificity: While Poland is the focus, explicitly state that findings may not be generalizable to cultures with vastly different views on death (e.g., East Asia, Middle East).

## 6 Conclusion

The study shows that medical professionals have different attitudes towards death. Paramedics (the oldest respondents) do not avoid thinking about the process (DA). Furthermore, they are the most convinced that death is a natural phenomenon (NA) and a release from earthly suffering (EA). Respondents from the paramedic group are less likely to avoid thinking about death than those from the doctors and nurses group, where this factor is significantly higher (DA). In addition, medical doctors and nurses believe that death is neither good nor bad (NA) and does not end all human problems (EA). In the study groups of physicians, nurses, and paramedics, no significant differences were found in terms of distance from death anxiety (FD), with the exception of young female nurses, who reported a higher death anxiety than young female medical doctors. Paramedics, compared to nurses, demonstrate a higher spiritual experience of death (AA) and generally do not want to think about death (DA). They are also the most reserved in avoiding death (EA), viewing it as a natural process of the end of human life. Looking at the data obtained in general, it can be concluded that paramedics, as the oldest respondents and the most professionally experienced, show strong attitudes towards death and are familiar with the process of dying. The presented research also showed that, in addition to the respondent’s profession, the age and gender of the respondents also influence the survey results. These findings suggest the implementation of comprehensive educational and training programs tailored to the specific needs of healthcare professions to better manage attitudes and emotional responses related to death. For nurses, integrating death education into clinical training—such as courses on end-of-life care, palliative care workshops, and simulation exercises on dying—can promote healthier attitudes toward death, improve coping mechanisms, and enhance the quality of terminal care. For paramedics, specialized training in stress management and advanced communication skills can strengthen their constructive approach to death. Training programs for physicians could include modules focused on reflection and existential discussions to reduce avoidance behaviors and support holistic patient care. Ultimately, educational interventions that foster open dialogue about death, spiritual and existential reflection, and practical end-of-life care skills are essential. These programs should consider the diverse life experiences, ages, and professional backgrounds of healthcare workers, as well as gender differences in attitudes toward death, to enable the development of personalized support strategies.

## Data Availability

The raw data supporting the conclusions of this article will be made available by the authors, without undue reservation.
